# Dissecting stepwise mutational impairment of megakaryopoiesis in a model of Down syndrome–associated leukemia

**DOI:** 10.1172/JCI161659

**Published:** 2022-07-15

**Authors:** Edward J. Evans, James DeGregori

**Affiliations:** 1 Department of Biochemistry and Molecular Genetics and; 2 Linda Crnic Institute for Down Syndrome, University of Colorado Anschutz Medical Campus, Aurora, Colorado, USA.

## Abstract

Individuals with Down syndrome (DS) have more than 100-fold increased risk of acute megakaryoblastic leukemia (AMKL), but its pathogenesis is poorly understood. In this issue of the *JCI,* Arkoun et al. engineered stepwise DS-AMKL–associated mutations in *GATA1*, *MPL*, and *SMC3* in human induced pluripotent stem cell (iPSC) clones from individuals with DS to dissect how each mutation affects gene expression control and megakaryocytic differentiation. The authors showed that the mutations cooperatively promote progression from transient myeloproliferative disorder to DS-AMKL. This study highlights the importance of mutation order and context in the perturbations of transcriptional and differentiation pathways involved in the evolution of hematologic malignancies, which will be critical for the development of preventative and therapeutic interventions.

## Acute megakaryoblastic leukemia of Down syndrome

Down syndrome (DS) is the result of trisomy of chromosome 21 (T21). Children with DS have a heightened susceptibility to many hematologic malignancies, especially leukemia (1, 2). Notably, 5% to 10% of children with DS under four years of age are diagnosed with the preleukemic state of transient myeloproliferative disorder (TMD), which is invariably driven by *GATA1* mutations generating a protein with a truncated N-terminus (GATA1s). Fortunately, in most of these children, the TMD and the associated *GATA1*s mutation spontaneously resolve with age (3–5). However, 20% to 30% of these patients with TMD acquire additional mutations and progress to acute megakaryoblastic leukemia (DS-AMKL). The additional mutations occur in cohesin family genes, the CTCF gene encoding a key regulator of chromatin architecture, genes encoding members of the polycomb repressive complex–2 (PRC2), including EZH2, and components of cytokine signaling, such as JAK2 or MPL (6–8). To help understand the evolution of TMD to DS-AMKL, it is necessary to develop human-relevant models that can recapitulate the DS-AMKL mutations and allow examination of progressive perturbations of megakaryocytic differentiation and other disease phenotypes. In this issue of the *JCI*, Arkoun and colleagues accomplish this objective using a stepwise technique to introduce *GATA1*, *MPL*, and *SMC3* mutants into induced pluripotent stem cells (iPSCs) from humans with or without DS (9). The researchers uncovered the individual contributions of each variant and how they could cooperate with T21 to lead to DS-AMKL.

The authors generated 20 different disomic and trisomic iPSC clones harboring a combination of *GATA1s*, *MPL^W515K^*, and heterozygous loss of *SMC3* (*SMC3^+/–^*) using CRISPR/Cas9 technology for stepwise gene editing and validated these changes with functional assays. MPL is a transmembrane receptor for thrombopoietin, which is necessary for megakaryocyte maturation into platelets. The intracellular domain mediates signaling through interactions with JAK2. Multiple *MPL* gain-of-function amino acid substitutions at position 515 result in myeloproliferative disorders through thrombopoietin-independent activation of the JAK/STAT pathway (10). Interestingly, W515K/L mutations have also been seen in both AMKL from people with T21 and in leukemias from euploid individuals (D21) with somatic acquisition of an additional chromosome 21, potentially contributing to altered megakaryocytic differentiation (7, 11). *MPL* mutations in the context of T21 and *Gata1*s are sufficient to induce megakaryocytic leukemia in mice (12). Additionally, the authors hypothesized that haploinsufficiency of the cohesin gene *SMC3* through heterozygous inactivation would alter chromatin accessibility of GATA1s binding and consequently alter the transcriptional control of megakaryocytic differentiation. Given that these mutations individually contribute to disrupting the myeloid lineage, the stepwise iPSC models that the authors constructed enabled the authors to parse how each mutation interacted with the others to result in the DS and DS-AMKL phenotypes.

## Cooperativity between mutants

**Figure 1:**
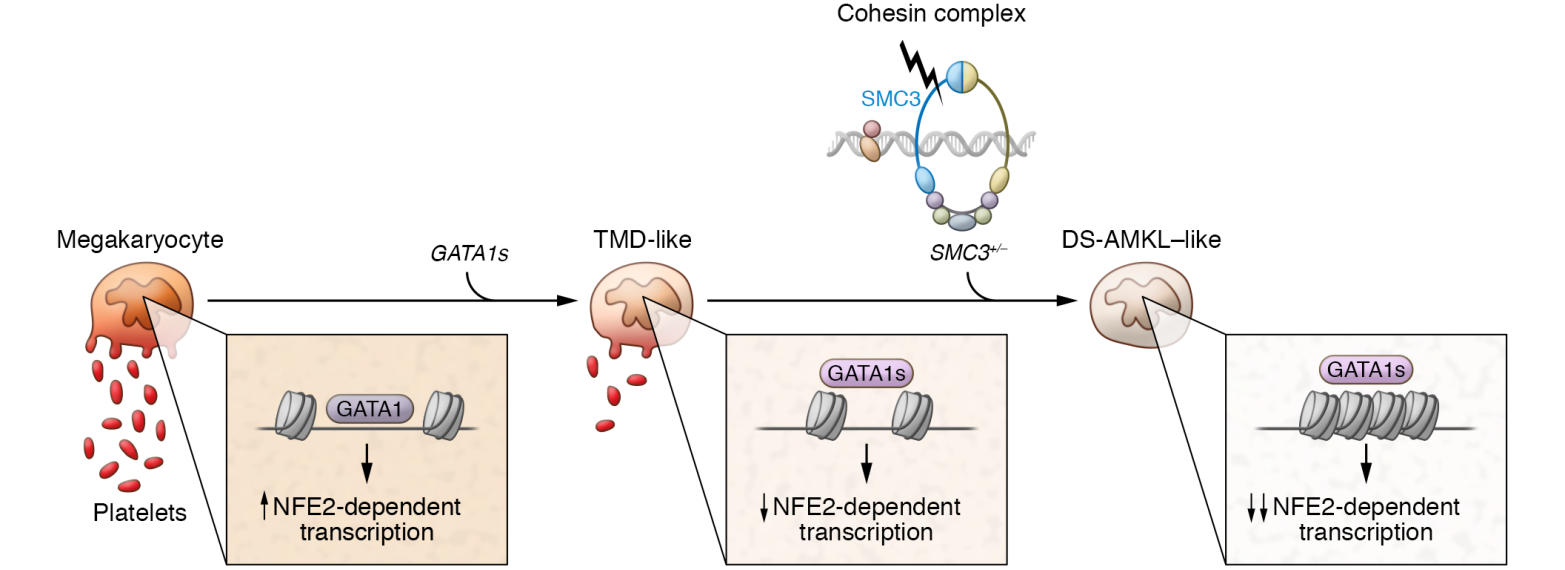
Progressive disruption in megakaryopoiesis in a model of DS-AMKL. The *GATA1s* mutation in hematopoietic progenitors induced in T21 iPSCs impairs megakaryopoiesis and leads to inefficient platelet generation and a TMD-like state, at least in part mediated by reduced chromatin accessibility and expression at NFE2 target genes. Subsequent inactivation of the cohesion complex component SMC3 (*SMC3^+/–^*) further reduces chromatin accessibility and expression of NFE2 target genes, leading to impaired platelet formation and the progression of a more AMKL-like state.

There are two major strengths of the Arkoun et al. study. First, the authors started with hematopoietic progenitors derived from human iPSC cells generated from individuals with T21 or D21, contrasting with many previous studies using mouse models. Second, as mutations were generated in a stepwise fashion, the effect of individual mutations on cellular phenotypes could be compared with that of those in combination, providing insight as to how these mutations cooperate to lead to DS-AMKL. Using RNA-Seq for gene expression, ATAC-Seq for chromatin accessibility, and staining of α-granules and lysosomes for assessment of megakaryocytic maturation, Arkoun et al. showed phenotypic transformations at each step and how T21 enabled these changes. The authors showed that GATA1s with the addition of *SMC3^+/–^* increased MK progenitors, synergistically prevented proplatelet (PPT) formation through a disrupted demarcation membrane system, and mimicked gene expression signatures previously reported for the progression from TMD to DS-AMKL. These observations are consistent with a differentiation block, enabling the expansion of progenitors through self-renewal, a hallmark of many forms of leukemia. Notably, the *SMC3^+/–^* mutation had opposite effects on platelet maturation and NFE2 inhibition, depending on whether or not it was preceded by the GATA1s mutation. Thus, the order in which mutations were acquired affected phenotypes controlling progression to DS-AMKL. Through RNA-Seq and ATAC-Seq, the authors showed that GATA1s hindered the NFE2 transcriptional pathway through reduced chromatin accessibility, with the subsequent *SMC3^+/–^* mutation enhancing these effects (Figure 1). Importantly, ectopic expression of NFE2 partially restored megakaryopoiesis. NFE2 is a member of the Cap’N’Collar transcription factor family that regulates megakaryocytic maturation and platelet production and is important for oxidative stress response (13). Given that people with DS have increased reactive oxidative species and defective mitochondria (14), inhibition of the cytoprotective NFE2-regulated transcriptional program upon mutation of GATA1s (causing TMD) could provide additional pressure for further disease evolution. The mechanism by which cohesin mutations contribute to leukemogenesis has been elusive, and the Arkoun et al. study now provides a model through altered transcriptional control of key differentiation pathways. Future investigations will hopefully flesh out how cohesin disruption interacts with GATA1s mutation to further impair chromatin accessibility at these genes and thus promote TMD to AMKL progression.

## The influence of the T21 context

The fact that GATA1s-driven TMD is apparently unique to individuals with DS (7) and that the prevalence of AMKL is substantially higher in people with DS highlights the importance of T21 in the onset of these hematologic malignancies. AMKL is much rarer in children without DS, and these cases of AMKL rarely exhibit the GATA1s mutation (7, 8). Notably, in some of these non-DS-AMKLs, somatic gain of chromosome 21 is observed. Other differences are apparent, including the frequency of mutations in cohesin complex components, occurring in about half of DS-AMKL, but less commonly present in non-DS-AMKL (approximately 10%) (7). Patients with non-DS-AMKL often exhibit chromosomal rearrangements that generate fusion proteins not seen in DS-AMKL. One could speculate that these alterations contribute to the impaired megakaryocytic differentiation otherwise imparted by GATA1s mutation in DS-AMKL. Intriguingly, instances of non-DS-AMKL that lack these fusion proteins frequently exhibit mutations in GATA1 and are more likely to have cohesin mutations (15). Thus, DS-AMKL and non-DS-AMKL may represent convergent evolution to a similar disease through distinct mutational routes.

A recent study from Wagenblast and colleagues, leveraging CRISPR to engineer DS-AMKL mutations in human CD34^+^ fetal hematopoietic progenitors from individuals with and without DS, demonstrated that inducing GATA1s in T21 (but not D21) progenitors leads to a TMD-like disease, even though GATA1s with a mutated cohesin gene can result in AMKL in both D21 and T21 contexts (16). T21 hematopoietic progenitors exhibit reduced clonogenic capacity relative to D21 progenitors, and GATA1s mutation restores clonogenicity selectively in the T21 background, suggesting that GATA1s mutation only enhances progenitor fitness in the T21 context (16). Of course, since subsequent mutations need to happen in the *GATA1s*–mutated background, the expanded GATA1s TMD clone (the target for the next mutation) promoted by the T21 context greatly increases the odds of progression to AMKL. Importantly, the Wagenblast et al. study also established that *GATA1s* mutation only engenders the preleukemic state in fetal or newborn hematopoietic progenitors, providing an explanation for why the window for DS-AMKL genesis is limited to very young children.

Arkoun et al. also recognized the importance of comparing isogenic disomic clones with mutations analogous to the trisomic models. Transcriptome analysis showed enrichment of the MYC and MYB proliferation pathways in *GATA1s/MPL^W515K^/SMC3^+/–^* (GMS) combined mutant clones in isogenic T21 clones relative to their D21 counterparts, consistent with greater proliferative and clonogenic potential when these mutations were engineered in the T21 background. Accordingly, the authors demonstrated that a MYC inhibitor caused greater reductions in the megakaryoblast proliferation in a T21-GMS context relative to D21-GMS, providing a targetable pathway for DS-AMKL and highlighting the value of investigating multistep carcinogenesis in different genotypic contexts.

## The need for multiple models and next steps

It is worth emphasizing that Arkoun et al. constructed their model to specifically investigate megakaryocytic differentiation and proliferation in the DS context. While noting the consistency of their results with other published models of DS-AMKL, particular deviations serve as reminders of the limitations and the difficulties of fully recapitulating phenotypes observed in DS-AMKL. As noted by the authors, their iPSC model did not generate a leukemic state amenable to serial transplantation, which is commonly used to showcase self-renewal, a hallmark of blood cancers. Additionally, while the in vitro model provides a powerful tool for molecular dissection, such a system lacks the extrinsic microenvironment present in vivo. Given that people with DS have increased inflammation (17), including hyperactive interferon signaling (18), and the tight interplay between inflammation and megakaryocyte and platelet maturation and function (19), in vivo studies of TMD and AMKL, whether in mouse models or by using human samples, could provide additional insight into DS-AMKL evolution.

Like many good studies, Arkoun et al. generates numerous intriguing questions and future directions. (a) Are there distinctions in megakaryocytic phenotypes in DS-AMKL with different cohesin gene alterations, and how do they interact with GATA1s and T21? (b) What are the cell-extrinsic factors that could influence the deleterious impact of AMKL mutations on megakaryocytic maturation, and are those factors T21-dependent? (c) How would additional mutations in the PRC2 complex, such as in EZH2, influence the transcriptional control of megakaryocytic differentiation and its alteration during AMKL development? (d) And perhaps most importantly, can an enhanced understanding of TMD and DS-AMKL evolution elucidate interventions to reduce progression (particularly at the TMD stage) or, better, to eliminate the disease? Hopefully, questions like these can be addressed in the future to better understand perturbations of megakaryopoiesis in people with DS and highlight therapeutic options to mitigate the prevalence and burden of DS-AMKL. With advancements in technology and pervasive use of multiomics approaches, we have the tools and opportunity to reduce the impact of TMD and AMKL on kids with DS.

## Version 1 07/15/2022

Electronic publication
